# The Remedy and Its Dose

**Published:** 1883-08

**Authors:** G. H. G. Jahr


### THE REMEDY AND ITS DOSE.

Dr. G. H. G. Jahr.

Psychical (as well as physical) diseases may be cured with all degrees of *attenuation* from 1 to 1,000, or even $\phi$, in drops, or with globules either dry on the tongue or dissolved in water, for, after all, the chief matter is always the *right choice* of the *remedy*, not its *dose*. But though we may achieve our object equally as well with small doses seldom repeated, yet it is well established that medicines prescribed in *too* large and *often-repeated* doses *must* be hurtful to those to whose symptoms the remedy is unsuitable, hence it still further proceeds from this that the practitioner, so long as he has not yet ascertained the appropriateness of the remedy to the symptoms of the case by actual experiment, will always do better if he exhibit the remedy at the commencement, experimentally, in an extremely small dose, and only when this appears to be followed by a beneficial change, ever so slight, to prescribe the continued use of the medicine in repeated doses.* Then if the remedy is really suitable there are no degrees of attenuation and size of doses so small that it would not bring to light the efficacy and show the appropriateness of the remedy by *some* sort of sign of incipient improvement.

Great attention and power of observation on the part of the physician are very necessary for the recognition of the first indications

---

* Hering advised us to *stop* the remedy when any beneficial change was apparent. And only to continue it when such improvement seemed to have ceased.—Ed.

of improvement; and as this is not the case with every one, it is, therefore, easily conceived how often one may hear it asserted that this or that remedy produced great improvement in large, massive doses, but in small doses none, not even the slightest. Such a case has never occurred in practice, and never, even when it was necessary for the continuance of improvement already begun that repeated copious doses were given, have we seen with an *appropriate* remedy that the first dose, however small, *did nothing at all.* In the majority of cases, not only of psychical, but also of other diseases, we perceive in the first twenty-four (in acute diseases much earlier, in chronic cases in two or three) days at least *some* signs by which we can judge whether the remedy answers or not, whether there will be aggravation or improvement, and are able to assure ourselves of its appropriateness or inappropriateness, and arrange our further treatment accordingly. All that we can recommend in this respect is limited to *one rule,* which, however, we recommend equally to every one and in every case: *Do first of all your utmost, with the assistance of a thorough examination of the patient, to discover every conceivable symptom and phenomenon which may serve as indications for the choice of a remedy. Search then* for that remedy whose special actions agree most nearly with these signs; *prescribe the* smallest dose of it at *first, experimentally,* and when you have accomplished this OPEN YOUR EYES WIDE AND OBSERVE YOUR PATIENT! You will thus soon see what is further to be done, whether the remedy must be continued and given in more frequent and larger doses or exchanged for another more suitable. To furnish any other rule, according to which young practitioners require merely to look into a formulary, as into a pocket recipe-book, in order to find for each case a dose indicated, which they may prescribe without consideration and then go their way, and without troubling themselves any further about their patient beyond merely feeling the pulse—is a clear impossibility.

He who cannot observe and knows not how to help himself, is not made for a physician, and for him no one can give any suitable rule whatever.—*Jahr's Mental Diseases.*

